# Food Security and Diet Quality Improvements among At-Risk, Low-Income, Older Adults following a Free Meal Pilot Program

**DOI:** 10.3390/ijerph21030344

**Published:** 2024-03-14

**Authors:** Makenzie Barr-Porter, Kendra OoNorasak, Tammy Stephenson, Ryan Goodson, Sofia Bonilla, Abraham Alhamdani

**Affiliations:** 1 Department of Dietetics and Human Nutrition, University of Kentucky, Lexington, KY 40506, USA; kendraoonorasak@uky.edu (K.O.); tammy.stephenson@uky.edu (T.S.); 2 College of Health Sciences, University of Kentucky, Lexington, KY 40506, USA; ryan.goodson@uky.edu; 3 College of Nursing, University of Kentucky, Lexington, KY 40506, USA; sofia.bonilla@uky.edu; 4 Department of Neuroscience, University of Kentucky, Lexington, KY 40506, USA; abraham.alhamdani@uky.edu

**Keywords:** food security, diet quality, low income, older adult, meal program

## Abstract

Older adults are at a greater risk for food insecurity compared to the general population. This study aimed to describe changes in diet quality and food insecurity following a free meal program at low-income, older adult housing complexes. Study participants were recruited from two low-income older adult public housing complexes in Kentucky. Fifty participants were enrolled and received 1–4 free weekly meals across 3 months as part of the Meals on Wings pilot program. Surveys and a 24 h dietary recall were completed at baseline and 3 months. Participants were predominately female, 69 years of age, and Black or African American race. Food security status (FSS) and dietary quality through the Healthy Eating Index (HEI) were assessed as primary measures. Participants were placed in “at-risk” categories of the (1a) lower quartile of the HEI and (1b) lower than 51% HEI, or (2) “low” or “very low” food security status based on the USDA Household Food Security Screener (FSS). Multiple linear regression (MLR) models were conducted for HEI and FSS scores to include time, group, time*group interaction, and control of meal utilization percentage (i.e., dose). The MLR for the HEI quartile had a significant time by quartile interaction that was present for an increase in the HEI in Q1 (*n* = 12; 32.42 ± 5.95% to *n* = 6; 46.10 ± 10.62%; *p* < 0.0001) and a decrease in the HEI for Q4 (*n* = 12; 70.68 ± 7.13% to *n* = 9; 52.36 ± 11.57%; *p* < 0.0001). For those low food security participants (*n* = 24; 48.0%), the average food insecurity score from the 6-item USDA screener improved from 4.09 ± 1.62 at baseline to 2.63 ± 2.41 at 3 months (*p* = 0.0064). The MLR for the FSS had a significant group*time interaction (*p* = 0.0071). In our population, particularly those vulnerable with lowest dietary quality and food insecurity status, we did see improvements across the free meal pilot program. However, a small sample, limited generalizability, and limited data collection measures urge caution when using these results to extrapolate for the general population. However, the current results are promising and should encourage further investigation of the effects of meal assistance programs on the health and well-being of older adults.

## 1. Introduction

The United States (U.S.) population is aging at a rapid rate. In the past decades, the number of older Americans has increased five times faster than the total number of Americans [1]. In 2021, almost a quarter of the U.S. population were older adults aged 60 years or older, outnumbering all American children and teens under 18 years [1]. The U.S. population continues to age; the older American population is expected to double and become an even larger portion of the population by 2050 [2]. Between 2010 and 2020, the median Kentucky adult age increased from 38.1 to 39.4 in the state [3]. In 2019, there were more than a million Kentuckians aged at least 60 years, representing almost a quarter of the total Kentucky population [4]. This number is likely to continue increasing, largely due to the peak of the baby boom generation in 1957.

The aging of the U.S. population presents a unique consideration regarding health JUand well-being. Detrimental health issues typically arise and exacerbate as one ages; thus, adults aged 60 years and older experience higher rates of frailty and pre-frailty that are related to falls, chronic diseases (e.g., diabetes, cardiovascular diseases), and mortality [5,6]. Furthermore, older adults are at risk of facing cognitive decline, which can begin at varying times with two-thirds of people developing some level of cognitive impairment by age 70 [5]. In Kentucky, almost half of Medicare beneficiaries have four or more chronic health conditions and 36% of older adults live with a disability. Consequently, 11.4% of older Kentuckians experience falls with injuries [4]. For many older adults, these health declines can dramatically affect one’s ability to function independently [6]. There are also subsequent impacts on the well-being and social connections of older adults. Nearly one-fourth of older adults are considered socially isolated and experiencing loneliness, exacerbated by circumstances brought about by aging [7]. These experiences are heightened in at-risk populations, especially aging communities that are low-income, underserved, and racially minoritized [8,9].

Healthcare expenditures have financially burdened many older adults with individuals aged 65 and above spending an average of USD 12,411 each in healthcare costs in 2018 [10]. Several older adults are at risk of experiencing at least one year of poverty at some point after entering this population with race and education affecting this risk [8]. Older Kentuckians are especially impacted by this with 12.6% living below the federal poverty line and half of single older Kentuckian households lacking financial means to pay for basic needs [4]. In 2021, Kentucky was ranked one of the worst four states in the nation for older adults at risk of and facing hunger. A total of 14.6% of the Kentucky population fell into this category, with food insecurity rates highest among those 55 years or older [3,11]. Though meeting daily dietary recommendations is critical for optimal older adult health and well-being, such as consuming five or more servings of fruits and vegetables, less than 30% of older Kentuckians meet these recommendations [4]. The food insecurity faced by older adults is not only the result of these individuals sitting at a low socioeconomic status, however. It is a multifaceted issue that can affect older adults for many reasons. For example, several older adults lack the relevant cooking and or nutritional knowledge to prepare healthy meals [9].

Partly due to the alarming prevalence of unaddressed food insecurity, the older population is at risk of malnutrition and impaired cardiovascular health [12,13,14,15,16]. Malnutrition is often under-recognized, even though it is associated with increased hospitalization, healthcare costs, and disease burden among older adults [17]. Federal and state food and nutrition assistance programs, such as the Meals on Wheels America Program and Supplemental Nutrition Assistance Program (SNAP), target malnutrition by increasing healthy food access and offering supplemental food assistance and/or monetary contributions to improve health outcomes in low-income populations [4]. However, limited evidence exists regarding the impact of these programs and interventions on older adults’ dietary quality and health outcomes. Therefore, it is becoming increasingly important to examine the effectiveness of these assistance programs in improving the health and well-being of older adults affected by or at risk of chronic conditions. This study aimed to describe changes in diet quality and food insecurity following a free meal pilot study program at low-income, older adult housing complexes in an urban setting in central Kentucky.

## 2. Materials and Methods

The study was conducted at one land-grant university in the United States in collaboration with community partners in the central Kentucky area. The protocol was conducted in accordance with the Declaration of Helenski, and the protocol received expedited review and approval at the University of Kentucky Institutional Review Board (#86433). The overarching program this study focuses on, Meals on Wings, is an initiative that utilizes local hospital partnerships to recover leftover foods and repurpose those foods into healthful meals for local food-insecure older adults. This pilot study aimed to initiate the program in central Kentucky, form partnerships with local hospitals and low-income senior housing, and begin a small service of meals. Meal ingredients were acquired from the area hospital for recovering to our university’s student-run Campus Kitchen at the University of Kentucky (CKUK) organization and repurposed into meals for the community [18]. The CKUK is a student-led organization that aims to reduce food waste in the surrounding area and divert those left over foods into repurposed, healthful meals for the community. Foods from the Meals on Wings program were recovered, transported, and repurposed in the CKUK by student leaders multiple days per week throughout these 3 months. Once meals were prepared by these trained CKUK student leaders, they packed meals, maintained food safety practices and holding temperatures, and delivered meals to the participant housing sites.

The study utilized a pre-/post-study design to examine changes in dietary quality, health behaviors, and food security of older adults after receiving free meal assistance across three months. For this pilot program, hot and cold meals were available for residents to receive for up to 4 meals per week for up to 50 participants. Whether participating in the study or after removing themselves at any time, all individuals were still able to receive meals.

### 2.1. Researcher Training

Data collection measures were overseen by lead researchers on the project. To ensure data were collected in an effective manner, 7 student research assistants were trained to perform data collection measures (height and weight). Research students (1 graduate student, 6 undergraduate students) were trained in one 3 h training session that included demonstration by the lead researcher, a practice session, and inter-rater reliability (IRR) testing. Inter-rater reliability included performing measures on two practice participants to ensure reliability of student researcher measures to 80%. Once IRR was confirmed between all student researchers, data collection began.

### 2.2. Participant Recruitment and Eligibility

Study participants were recruited from two low-income older adult public housing complexes in Kentucky. The study sites were recruited through local email listserv requests for interest in participating as a partner in the research study. Four sites expressed interest. Two sites, with which we have established relationships through other community outreach projects, expressed interest and had the capacity and willingness to support this project and research study.

Participants were recruited via word of mouth, in-person, by the research team and housing complex staff in early May 2023. Program eligibility included participants being 60 years of age or older, able to speak and understand the English language, reside in one of the two housing complexes, ability to complete physical assessments and stand on their own, and deemed cognitively aware using the 6CIT measure [19]. If ineligible to participate in the study, individuals were still able to receive meals, but data were not collected for the study. Only those eligible and completing baseline data collection were included in this pilot study sample.

### 2.3. Data Collection Measures

Once deemed eligible to participate, and at 3 months post, individuals were physically measured for height via Seca stadiometer, and weight and body mass index (BMI) via Tanita electrical impedance scale. Each measure was taken twice and averaged. Averages were used for analysis.

Additionally, behavioral surveys were given at baseline and 3-month intervals. Demographic variables, including the Food Security Screener (FSS), were assessed by the 6-item United States Department of Agriculture’s Household Food Security Screener [20]. Scores were calculated and summed. FSS scores 0 and 1 were classified as high/marginal food security, scores 2–3 were classified as low food security, and scores 4–6 were classified as very low food security. The 5-item sarcopenia assessment (SARC-F) was used to measure Strength (S), Assistance walking (A), Rising from a chair (R), Climbing stairs (C), and Falls (F) on a scale of 0 to 10, with higher scores indicating higher risk [21]. Mini Nutritional Assessment (MNA) short form measured risk for nutrition support with 6 questions including self-reported height and weight, recent sickness, or recent unintentional weight loss. Screening scores range from 0 to 14 points with lower MNA-sf scores indicating malnutrition risk [12]. The World Health Organization (WHO) Well-Being Index assessed 5 statements of overall well-being in the last two weeks on a 6-point Likert scale from “all of the time” (score of 5) to “no time” (score of 0) with items including “I have felt cheerful and in good spirits” or “I woke up feeling fresh and rested” [13]. Scores are summed for all 5 items from 0 to 25 and multiplied by 4 to have a final score range from 0 to 100. Lower WHO Well-Being scores indicate a lower quality of life. Finally, loneliness was measured on the UCLA 3-item Loneliness Scale with a scale including “hardly ever”, “some of the time”, and “often” and included statements such as “how often do you feel left out?” Loneliness scores range from 3 to 9 with those scoring between 6 and9 indicating loneliness [14].

To examine dietary quality improvements, 24 h dietary recalls were administered via the National Cancer Institutes (NCI) Automated Self-Administered-24 (ASA-24) platform [22]. ASA-24 was delivered online and older adult participants were assisted with their 24 h dietary recall by trained research staff. ASA-24 was collected once per time point, baseline and 3 months. Following data entry, ASA-24 results for baseline and 3 months were assessed in SAS software to calculate Healthy Eating Index-2015 (HEI) scores, based on the most recent available NCI SAS code. HEI scores range from 0 to 100% of meeting the Dietary Guidelines for Americans (DGAs) with lower scores indicating diet not adequately meeting the DGAs.

### 2.4. Data Analyses

Descriptive variables were analyzed for frequencies of categorical variables and measures of central tendency for continuous variables. To assess baseline to 3-month changes in continuous variables (HEI, FSS, MNA, WHO, Loneliness), matched pairs *t*-tests were performed. To further examine differences among participants scoring particularly low on HEI and as food insecure on the FSS at baseline, subsamples of those “at-risk” were examined. At-risk subsamples were formed by (1) HEI scores that were based on both (1a) scores between 0 through <51% and ≥51% and higher, and by (1b) quartiles from the full cohort and (2) FI status of low or very low. Multiple linear regression analyses were conducted on HEI and FSS scores with time, group, and group*time interaction terms as covariates. Pairwise comparisons of group by time interactions were completed. Finally, meal utilization/attendance (number of meals taken by participant/number of meals possible to obtain * 100) was used as a covariate in a subsequent, duplicate multiple linear regression to understand effect of dose.

## 3. Results

### 3.1. Full Cohort Descriptive Characteristics

Baseline descriptive characteristics (Table 1) of the population were collected at the initial time period for all enrolled. Participants (*n* = 50) enrolled in the study were predominately female, approximately 69 years of age, Black or African American, lived alone at home, had a high school degree and had an annual income between USD 0 and 30,000. Among health behaviors and assessments, participants were predominately in the overweight or obese categories of BMI (38; 84.4%).

Participants predominately self-reported the 6-item FSS as high/marginal food security (52.0%) (Table 2). Among behavioral-based survey scores, participants’ WHO Well-Being scores indicated an above average quality of life (62.48 ± 25.93). Participants’ average HEI score indicated approximately 50% of their diet meets the DGAs. Additionally, a separate measure indicated a lower risk of sarcopenia via SARC-F (score less than 4). Furthermore, participants also reported mild loneliness (4.56 ± 1.64), and the MNA-sf placed them, on average, in the “at-risk” category for malnutrition (10.93 ± 2.69). No significant changes were seen among screening tools from baseline to 3-month’s post-program for the full cohort, except for the FSS category (*p* = 0.0006).

For the full cohort, when assessing changes from baseline to 3 months in behavioral-based survey scores, there were no significant changes among the matched pairs *t*-tests for any of the survey tools (FSS, WHO Well-Being, HEI, SARC-F, UCLA Loneliness, and MNA-sf).

With free weekly meals available to each participant to receive across 15 weeks, for a total maximum range possible of 26–58 meals across the program duration, the average amount of meals received by participants was 20.83 ± 14.62 meals. Of the number of meals available per participant, the average percentage of utilization of the program was 58.52 ± 30.25%.

### 3.2. Healthy Eating Index

To assess changes among those most at-risk, subsamples were created from FSS and HEI scores to assess baseline to 3-month changes among the risk ranges of the screener tools. Of the cohort, 30 participants (60.0%) had a HEI score below 51% of the Dietary Guidelines for Americans, placing them “at-risk”, with an average of 41.54 ± 8.74%. In a paired sample *t*-test, there was not a significant difference between the baseline and 3-month scores by group (t(33) = −1.37, *p* = 0.1785). However, there was indication of a significance within pair interactions (*p* = 0.0128). From baseline to 3-month HEI scores, there was a significant time*group interaction (F(1,32) = 6.96, *p* = 0.0128). Pairwise comparisons indicated significance among the higher HEI group (≥51%) with a significant decline in their scores from baseline to 3 months (−10.03 ± 15.07%).

To further detect differences in HEI score changes across the program, HEI was categorized into quartiles (Q1 (low HEI): 23.26–40.68; Q2: 40.69–49.88; Q3: 49.89–59.89; Q4 (high HEI): 59.90–84.48). Separate *t*-tests were performed on HEI scores by time for each quartile group. Both Q1 and Q4 had significant differences from pre- to post-study (*p* = 0.0233 and 0.0012, respectively). Increases in HEI within Q1 were (*n* = 12) 32.42 ± 5.95% to (*n* = 6) 46.10 ± 10.62%. Decreases in HEI for Q4 (*n* = 12) were 70.68 ± 7.13% to (*n* = 9) 52.36 ± 11.57%.

**Figure 1:**
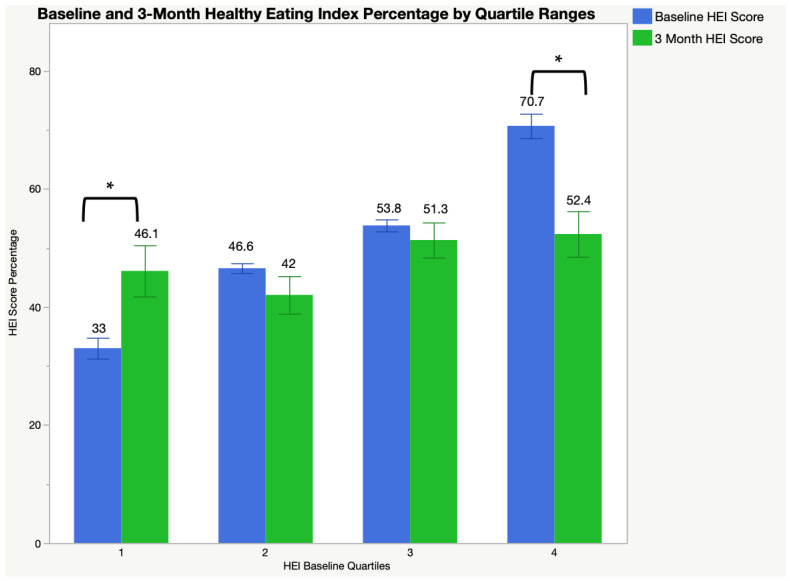
Healthy Eating Index scores from baseline and 3 months by quartiles. Healthy Eating Index (HEI) scores change from baseline and 3 months among quartile groups determined by baseline HEI scores (Q1 (low HEI): 23.26–40.68; Q2: 40.69–49.88; Q3: 49.89–59.89; Q4 (high HEI): 59.90–84.48). * Significant difference between baseline and 3-month HEI scores by quartile group; *p* < 0.05.

Multiple linear regression models were conducted to assess changes while controlling for meal utilization. Full HEI model for time, quartile, and quartile*time interaction was significant (F(8, 74) = 20.37; *p* < 0.0001, R2 = 0.71) (Table 3). In pairwise comparisons, those significant time*group interactions remained in Q1 and Q4 (both *p* < 0.0001) between the baseline and 3 months (Figure 1).

### 3.3. Food Security Screener

**Figure 2:**
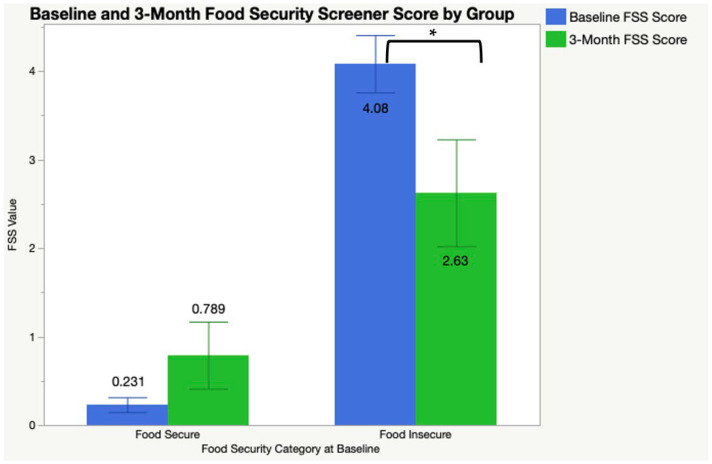
Food security scores from baseline and 3 months for at-risk group (low and very low food security status). Six-item food security screener (FSS) scores among an at-risk group (those with low/very low food security at baseline) at baseline and 3 months. * Significant difference between baseline and 3 month FSS scores by group; *p* < 0.05.

Additionally, 24 participants (48.0%) were classified as low or very low food security status at baseline and placed into a food insecure category. For those food insecure participants, the average food insecurity score from the 6-item USDA screener went from 4.09 ± 1.62 at baseline to 2.63 ± 2.41 at 3 months (t(15)= −3.16, *p* = 0.0064) (Figure 2). In a significant multiple linear regression model assessing FSS change from baseline to 3 months with time, group (low FSS and high FSS), Group*Time interaction, and controlling for meal utilization percent (F(4, 74) = 23.52, *p* < 0.0001, R2 = 0.57) (Table 3), there was a significant time * group interaction (*p* = 0.0047). Participants in the food insecure group had a significant improvement in score from baseline to 3 months (*p* = 0.0087).

## 4. Discussion

The current study aimed to describe diet quality and food security prior to, and following, a three-month free meal program among older adults in subsidized housing complexes. Following the intervention, no significant changes in health measures or behavioral survey tools were detected among the full cohort. Among a subsample of participants within a quartile of the group whose HEI scores were lower than 40.68% HEI scores, dietary quality scores on the HEI scale significantly improved, though on average, remained below 51% out of 100%. Likewise, among those with low or very low food security status, food security scores significantly increased following the free meal program but remained in the “low” food security range.

Though older adult participants in this study were low-income residents of subsidized housing, about half of the participants were considered food secure based on the USDA 6-item FSS screener. Similarly, in one 6-week county meal assistance program, half of the older participants were food secure [23]. This may indicate some discrepancies between low-income status and FSS defined and assessed by the current gold standard measures from the USDA. Due to federal and state social services for older adults as part of the Older Americans Act of 1965 [24], FSS may not be best determined by the current USDA FSS screener.

The average HEI score in our study was less than 51%, indicating that older participants had worse dietary quality compared to the average HEI score of 58–61% among the general American population [25]. Specifically, HEI scores among our lowest quartile significantly increased from baseline to 3 -months. Our findings are similar to a secondary analysis of the 2007–2016 NHANES data. The NHANES data compared nutrient intakes and diet quality of nationally representative U.S. older adults aged 60 years and older participating in the SNAP and those of other low-income, SNAP-eligible, older adults who were not participating in the program [26,27]. Specifically, total dietary quality (HEI score) for both SNAP older participants and non-participants from that secondary study was below 60%, which was considered a grade of F [26,27]. Though a secondary analysis study by Qin et al. found no significant difference in dietary quality of SNAP older participants and non-SNAP low-income older adults [28], our current study’s observed dietary quality scores improved from baseline to the 3-month follow-up among the lower quartile subsamples. However, interestingly, we also saw a significant decrease in HEI scores among the highest scoring quartiles. This finding could be due to singular dietary recalls taking place or self-reporting biases at baseline that over-reported healthy intake. Limitations are common among self-reported dietary intake; however, future work can aim to alleviate some of those issues. This is further discussed in this article’s limitations section.

Many studies have additionally found that food assistance programs like SNAP improve food security across one’s lifespan [29,30]. Particularly, this study’s findings on significant improvement of the food security score reveal a possibly viable approach for food assistance efforts to prioritize supporting older adults experiencing low and very low food security statuses. Among the SNAP-eligible population, those who were most at-risk nutritionally and those that were food insecure were least likely to meet recommendations but most likely to participate in SNAP [31,32]. Our findings indicate a need to focus on those particularly at-risk. With significant increases in the dietary quality scores on the HEI among the lowest quartile, and food security status among participants in “at risk” subsamples, an additional number of meals received through such meal assistance programs may elicit an observed improvement out of the “at-risk” and “low” score categories. Though this study had a short time frame, and was a pilot program, further investigation and validation measures are warranted to truly understand the program effect.

Though our findings did not indicate improvement in loneliness measures, some intervention studies collect baseline cross-sectional data only among older adults like FSS and social isolation levels without follow-up data [33]. Some studies that assess impacts of food assistance programs like Meals on Wheels have used a study design similar to ours, comparing baseline and follow-up data and finding significant improvements in outcomes, such as protein intake and malnutrition risk [34,35,36]. In terms of the scale of this study, the average number of meals distributed to older adults in the program by Bruce et al. (2022) was around 326 meals per week for 6 weeks, which was substantially greater than the amount the current study provided [37]. Being a pilot intervention study, there are opportunities to explore the sustainability and expansion of such intergenerational food assistance efforts [37].

Moreover, though an age requirement is often utilized to be eligible for governmental senior food assistance programs like Meals on Wheels [4], researchers believe it is critical to instead take a life course approach as part of health equity promotion efforts [38,39,40,41]. Fundamentally, food is a basic human right, and researchers urge the importance of shifting currently fragmented charitable food systems toward an equitable food system that sustainably nourishes all communities, regardless of race, age, or any socioeconomic background. This study’s results show the ways in which downstream solutions, such as free meal assistance, can impact nutritional quality, particularly for those at more risk of malnutrition and impaired health. These findings will also contribute to the limited literature on impacts on dietary quality of non-governmental older adult meal assistance programs.

### Limitations

The current study adds to the evidence base regarding health and food security among older adults, but the study is not without limitations. Although the population consisted of a relatively even spread of race and sex, several participants fell into the classification of high or marginal food security, not exclusively those with lower status. As we aimed to be inclusive to all, participants were able to self-select to participate in the research portion if meeting the eligibility criteria. Future work may benefit from further exclusion criteria to include and determine the impact of interventions on those most at-risk. Due to our purposive sampling, we are unable to determine external validity and may not have had robust representation for widespread generalizability.

Additionally, as this was a study with older adults, we aimed to reduce the participant burden of data collection periods. Due to this, we only collected data in a pre–post manner, and were unable to collect extensive and thorough measures such as multiple 24 h dietary recalls for validity. Another interesting item of note that should be taken into consideration with the results for future studies, is that meal utilization percentage (i.e., program dose) was not found to be a significant predictor of diet quality or food security status changes across the program period. These results suggest that more confirmatory variables should be included in future iterations of the project such as multiple dietary recalls, additional validated dietary survey measures, and a larger sample size to detect differences. In addition, capturing meals outside of the program (i.e., additional meals participants eat outside of the program provided meals) would be helpful. Because we only collected one 24 h recall, we cannot be sure of the intervention specific impacts. Likewise, among participants who consume a limited number of meals outside of the free program meals, the provided meals may be providing the bulk of nutrition throughout the week. Future iterations of projects such as these may consider controlling for the frequency of outside meals to understand the impact of these meal programs for those with limited access or availability to procuring their own food.

Finally, future work should consider the inclusion of mixed method approaches to capture further insight into day-to-day food environment influences. Further interactions with participants may allow for further inclusions of building a sense of community, understanding the needs of the population outside of meals, and building a capacity for expansion of an intervention such as this pilot program.

## 5. Conclusions

This study aimed to describe changes in older adult food insecurity and diet quality through a free meal pilot program. In our population, particularly those vulnerable with lower dietary quality scores and food insecurity status, we did see improvements in scores across the free meal program. Though these results are taken from a small sample size that cannot be generalized to wider populations, it serves as an addition to the literature surrounding food waste and food recovery efforts to divert leftover foods into repurposed, healthful meals. This pilot study aimed to describe these initial recovery meal efforts with older adults and identify areas of improvement for the study when scaling up. These current results are promising and encourage further investigation of the effects of meal assistance programs on the health and well-being of older adults. If the program continues to be effective for these individuals, it may serve as a model program for older adults across Kentucky and the U.S.

## Figures and Tables

**Table 1:** Descriptive characteristics of the population at baseline.

Descriptive Variable	
	*n* = 50
Sex	
Male	23 (46.0%)
Female	27 (54.0%)
Age	69.38 ± 7.00
Race	
Black or African American	25 (50.0%)
White	24 (48.0%)
Biracial	1 (2.0%)
Education Level	
Less than high school	8 (16.0%)
High school	20 (40.0%)
Some college	13 (26.0%)
4-year degree	9 (18.0%)
Income Level	
USD 0–30,000	49 (98.0%)
USD 31,000–60,000	1 (2.0%)
BMI (kg/m^2^)	
BMI Category	
Underweight	1 (2.2%)
Normal	6 (13.3%)
Overweight	12 (26.7%)
Obese Class I	8 (17.8%)
Obese Class II	11 (24.4%)
Obese Class III	7 (15.6%)

**Table 2:** Health and behavior measures at baseline and 3 months.

Measure	Baseline	3-Months	*p*-Value
	*n* = 50	*n* = 35	
FSS Score	2.08 ± 2.25	1.63 ± 2.21	0.2490
FSS Category			0.0006
High/Marginal	26 (52.00%)	23 (65.71%)	
Low	14 (28.00%)	6 (17.14%)	
Very Low	10 (20.00%)	6 (17.14%)	
WHO Well-Being Index	62.48 ± 25.93	64.57 ± 26.91	0.9765
HEI Score	50.85 ± 14.53	48.90 ± 10.49	0.1785
SARC-F Score	3.66 ± 2.53	3.71 ± 2.35	0.3788
UCLA Loneliness	4.56 ± 1.64	4.83 ± 1.85	0.3468
MNA-sf	10.93 ± 2.69	10.63 ± 2.55	0.7078

Matched pairs *t*-test for continuous variables (FSS, WHO, HEI, SARC-F, UCLA, and MNA-sf). Pearson chi square test for categorial variables (FSS category).

**Table 3:** Multiple linear regression for HEI and FSS groups.

	Explanatory Variable	Standardized Beta	Std Error	*t*	*p* Value	R Squared
HEI						
	Group					
	Q1	−0.5688	1.4806	−6.79	<0.0001 *	
	Q3	0.1455	1.3101	1.85	0.0693	
	Q4	0.6965	1.4769	8.45	<0.0001 *	
	Time	−0.1018	0.8220	−1.51	0.1356	
	Group*Time					
	Q1	0.4612	1.4741	5.54	<0.0001 *	
	Q3	−0.3391	1.3094	−0.69	0.4908	
	Q4	−0.0549	1.4443	−4.21	<0.0001 *	
	Meal Utilization %	−0.0408	0.0319	−0.60	0.5538	
						0.71
FSS						
	Group	−0.6716	0.1829	−8.49	<0.0001 *	
	Time	−0.1067	0.1819	−1.36	0.1790	
	Group*Time	0.2199	0.1818	2.77	0.0071 *	
	Meal Utilization %	0.0926	0.0068	1.18	0.2420	
						0.57

HEI Referent categories: Quartile 2 group and 3-month time. FSS referent categories: food secure group and 3-month time. * *p* < 0.01.

## Data Availability

Data available upon reasonable request to authors.
